# Autoimmune gastritis: a comprehensive review of pathophysiology, risk stratification, and management

**DOI:** 10.3389/fimmu.2026.1878128

**Published:** 2026-06-12

**Authors:** Minxiao Feng, Wenting Xu, Haiyan Zhu

**Affiliations:** 1 Department of Gastroenterology, Zhangjiagang TCM Hospital Affiliated to Nanjing University of Chinese Medicine, Jiangsu, China; 2 Department of Reproduction, Zhangjiagang TCM Hospital Affiliated to Nanjing University of Chinese Medicine, Jiangsu, China; 3 Department of Gastroenterology, The Third Affiliated Hospital of Zhejiang Chinese Medical University, Zhejiang, China

**Keywords:** autoimmune gastritis, familial aggregation, gastric neuroendocrine tumors, genetic susceptibility, risk stratification, thyroid autoimmunity

## Abstract

Autoimmune gastritis (AIG) is a chronic, organ-specific autoimmune disease characterized by the immune-mediated destruction of gastric parietal cells, leading to impaired acid secretion, vitamin B12 deficiency, and an increased risk of gastric malignancies. The diagnosis of AIG relies on endoscopic findings combined with serological markers and histopathological confirmation. This review synthesizes current knowledge on the pathophysiology, diagnosis, and management of AIG, with a special focus on familial aggregation, polyglandular autoimmunity, and emerging therapeutic strategies. We discuss the diagnostic challenges posed by serological variability, the complex interplay with Helicobacter pylori infection, and the diagnostic pitfalls of macrocytic anemia. Furthermore, we explore precision risk stratification models for gastric neuroendocrine tumors (gNETs) and gastric adenocarcinoma, emphasizing the roles of endoscopic surveillance and molecular biomarkers. Finally, we review emerging therapeutic options, including novel immunomodulators and microbiome-targeted interventions. This review provides a comprehensive framework for clinicians to navigate the complexities of AIG, from early diagnosis to long-term management, with the goal of improving patient outcomes and mitigating the risk of malignant transformation.

## Introduction

Autoimmune gastritis (AIG) is an organ-specific autoimmune disease characterized by the chronic, progressive destruction of gastric parietal cells in the corpus and fundus of the stomach (1–3). This immune-mediated assault leads to a cascade of clinical consequences, including impaired gastric acid secretion (hypochlorhydria or achlorhydria), reduced intrinsic factor (IF) production, and subsequent vitamin B12 malabsorption, which can ultimately results in pernicious anemia (3). The global prevalence of AIG is estimated to be around 3.85% (4), with a higher incidence in women and a significant association with other autoimmune conditions, particularly autoimmune thyroid disease (AITD) (5). The clinical significance of AIG extends beyond its direct effects on gastric function. Recent epidemiological studies have revealed that AIG is not merely a gastroenterological condition but rather a systemic autoimmune disorder with significant extraintestinal manifestations. The prevalence of AIG is substantially higher in certain geographic regions and ethnic populations, suggesting both environmental and genetic factors in disease susceptibility (4). Furthermore, the natural history of AIG demonstrates a progressive course, with early-stage disease often remaining asymptomatic and undiagnosed for years, only to manifest clinically when significant gastric atrophy and vitamin B12 deficiency have already developed (6). This delayed diagnosis underscores the importance of proactive screening in high-risk populations, including patients with established autoimmune thyroid disease, type 1 diabetes, or a family history of autoimmune conditions.

Historically, AIG has been underdiagnosed due to its often asymptomatic nature in the early stages, the nonspecificity of its clinical manifestations, and the insufficient awareness of this disease among endoscopists. However, with advancements in serological testing and endoscopic techniques, there is a growing recognition of its clinical importance. The diagnosis of AIG relies on endoscopic findings combined with serological markers and is confirmed by histopathological findings, including anti-parietal cell antibodies (PCA) and anti-intrinsic factor antibodies (IFA), and histopathological findings from gastric biopsies, which typically reveal chronic atrophic gastritis, intestinal metaplasia, and neuroendocrine cell hyperplasia (7). One of the most significant clinical implications of AIG is its association with an increased risk of gastric malignancies, including type 1 gastric neuroendocrine tumors (gNETs) and gastric adenocarcinoma (8). The chronic inflammation, achlorhydria, and hypergastrinemia create a microenvironment conducive to neoplastic transformation, necessitating long-term endoscopic surveillance and individualized risk stratification (9).

The epidemiology and clinical presentation of AIG demonstrate notable geographic and ethnic variations, reflecting both genetic predisposition and environmental factors. In Asian populations, particularly in East Asia, the prevalence of AIG has been reported to be higher than in Western countries, with studies from Japan and China documenting prevalence rates of 2-5% in certain regions (4). These variations may be attributed to differences in HLA allele frequencies, dietary factors, and the prevalence of H. pylori infection across different populations. Additionally, the clinical manifestations and disease severity can differ between ethnic groups, with some evidence suggesting that Asian patients may present with more advanced gastric atrophy at the time of diagnosis compared to their European counterparts (10). Furthermore, healthcare system differences, including the availability of endoscopic screening and serological testing, significantly impact the diagnosis and management of AIG. In countries with universal screening programs for pernicious anemia or autoimmune thyroid disease, AIG is detected earlier and more frequently, whereas in regions with limited healthcare access, the disease often remains undiagnosed until symptomatic complications develop (11). These epidemiological insights underscore the importance of culturally and geographically tailored diagnostic and management strategies for AIG.

The management paradigm for AIG has evolved significantly over the past decade. While traditional approaches focused primarily on symptomatic management and nutritional supplementation, contemporary practice increasingly emphasizes precision medicine strategies, including individualized risk stratification for malignancy and the exploration of immunomodulatory therapies aimed at halting or reversing the autoimmune process (12). This shift reflects a growing recognition that AIG, like other autoimmune conditions, may be amenable to disease-modifying interventions if identified and treated early in its course.

This review synthesizes the current understanding of AIG, from its immunopathogenesis to its clinical management, and highlights key concepts through illustrative clinical examples.

## Illustrative clinical examples of AIG

To illustrate the key clinical features of AIG, we briefly present three cases from a single family, all diagnosed with AIG and concomitant AITD. These cases represent original, unpublished clinical data from The Third Affiliated Hospital of Zhejiang Chinese Medical University. They are not adapted from previously published literature, but rather derive from our own clinical practice and are presented here for the first time. Written informed consent was obtained from all patients for the publication of their clinical and imaging data. These cases highlight the concepts of shared genetic susceptibility and familial clustering, consistent with the spectrum of autoimmune polyendocrine syndromes (13).

### Case 1 (Proband, 64F)

Presented with fatigue and mild anemia. Suspected AIG based on positive IFA and endoscopic/histopathological findings of atrophic gastritis, despite negative PCA. Concomitant AITD confirmed by elevated anti-TPO antibodies.

### Case 2 (First cousin, 74F)

Identified through family screening. Presented with iron deficiency anemia. Diagnosed with AIG based on positivity for both PCA and IFA, endoscopy and histopathology showed severe corpus-predominant gastric atrophy, pseudopyloric metaplasia and ECL hyperplasia. Concomitant AITD was also present.

### Case 3 (First cousin, 71M)

Asymptomatic, identified through family screening. Diagnosed with AIG based on positivity for both PCA and IFA. Endoscopy revealed corpus-predominant atrophy and pseudopyloric metaplasia. Concomitant AITD was confirmed by elevated anti-TPO antibodies and ultrasound findings.

**Figure 1:**
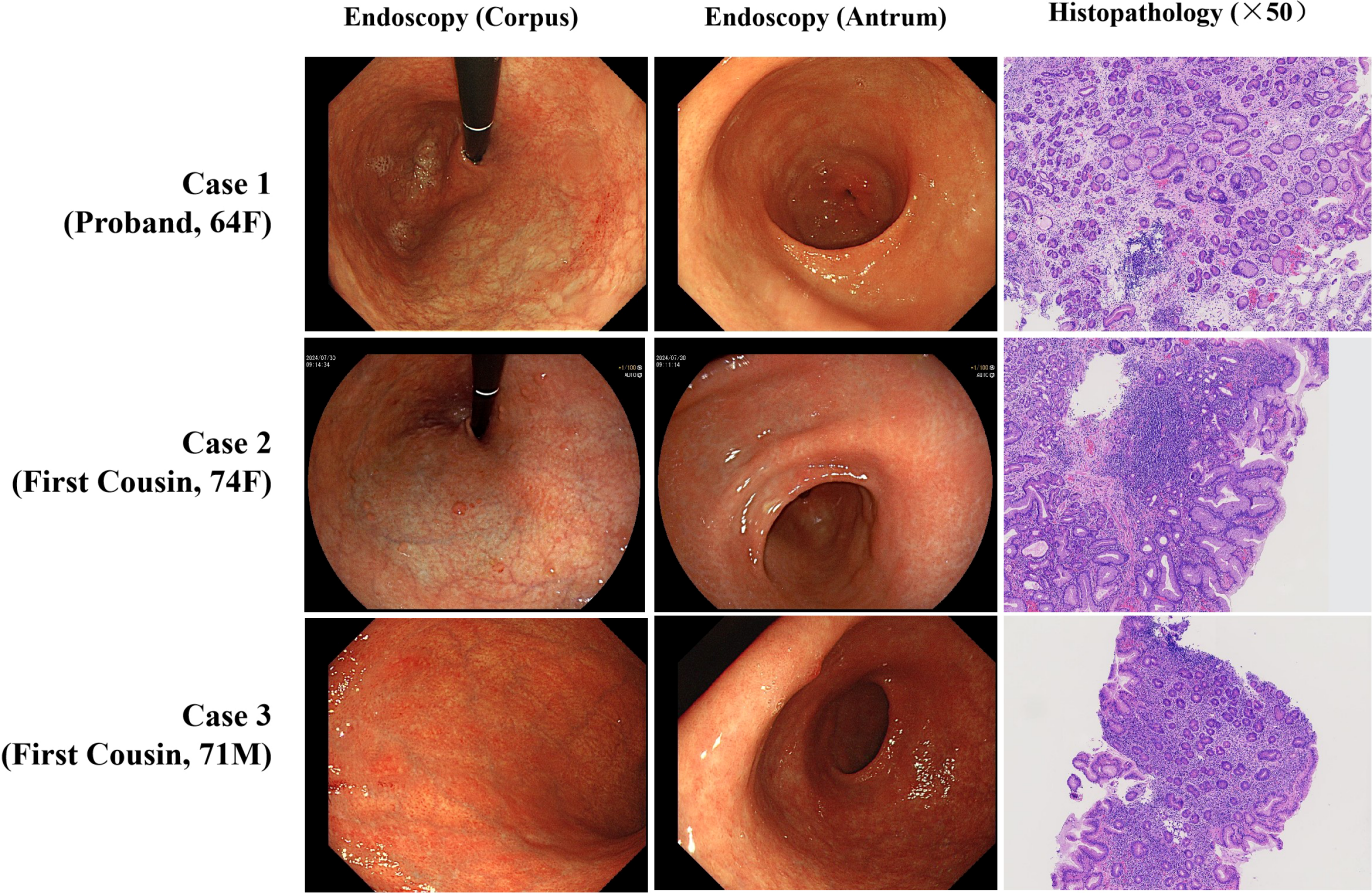
Representative endoscopic and histopathological findings of the three familial AIG cases. Each row corresponds to one patient. Left column: endoscopic images of the gastric corpus; middle column: endoscopic images of the gastric antrum; right column: histopathological examination (hematoxylin and eosin staining, ×50). Case 1 (Proband, 64F): Endoscopy reveals marked corpus mucosal atrophy with clearly visible submucosal vasculature; the antrum shows mild atrophic changes. Case 2 (First Cousin, 74F): Endoscopy demonstrates corpus and fundic mucosal atrophy with visible submucosal vasculature and polypoid elevations of residual oxyntic mucosa; the antrum shows mild atrophy. Case 3 (First Cousin, 71M): Endoscopy shows loss of rugal folds along the greater curvature of the corpus with scattered erythematous patches; the antrum shows mild atrophy. Histopathology (all three cases): Full-thickness chronic inflammatory infiltrate with reduction of native oxyntic glands and pyloric gland metaplasia (pseudopyloric metaplasia), consistent with the diagnosis of autoimmune gastritis.

These cases underscore the variable serological presentations of AIG, the importance of family screening, and the frequent co-occurrence of AIG with other autoimmune disorders. The clinical presentation of these three cases illustrates several important diagnostic principles. Case 1 demonstrates the diagnostic challenge of seronegative PCA with positive IFA, which occurs in approximately 20-30% of AIG patients and highlights the importance of testing both antibodies (14). The presence of IFA alone is sufficient for diagnosis, as it has a specificity of 95-100% for AIG (15). Case 2 exemplifies the more typical presentation with dual antibody positivity (both PCA and IFA), which is associated with more extensive gastric atrophy and higher risk of gastric neuroendocrine tumors (16). The severe ECL hyperplasia noted on endoscopy in this case is a precursor lesion for type 1 gastric neuroendocrine tumors and warrants closer surveillance. Case 3 represents the asymptomatic presentation identified through family screening, which is increasingly recognized as an important clinical scenario. Familial clustering of AIG has been documented in approximately 5-10% of patients, with first-degree relatives having a significantly elevated risk of developing the disease (17) (**Figure 1**). The identification of AIG in asymptomatic family members allows for early intervention and prevention of complications such as vitamin B12 deficiency and gastric malignancy. The presence of concomitant AITD in all three cases underscores the concept of polyglandular autoimmunity and the need for comprehensive screening of multiple organ systems in patients with AIG.

## Pathophysiology and immunogenetics of AIG

**Figure 2:**
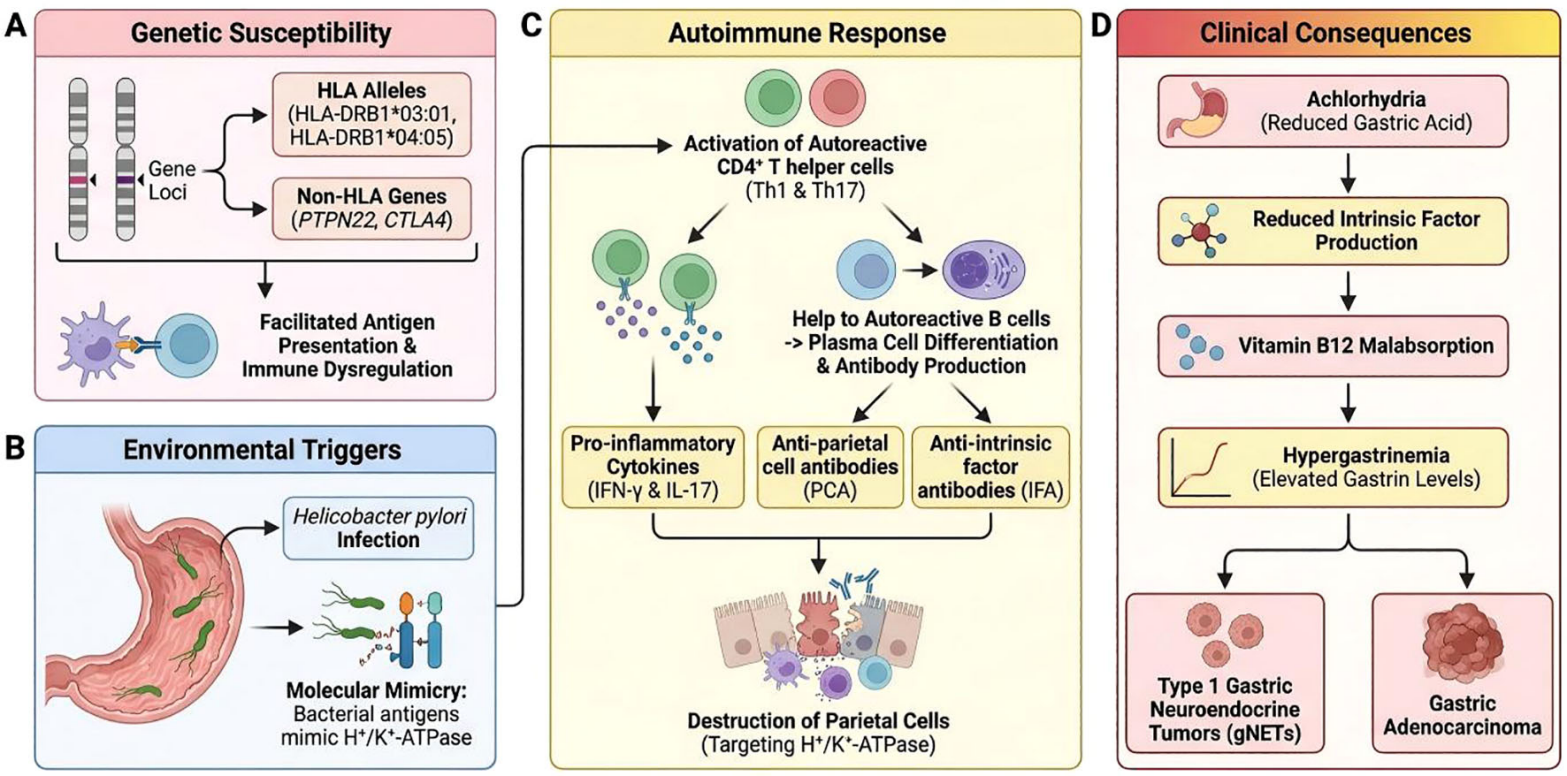
Pathophysiology of autoimmune gastritis. This comprehensive diagram illustrates the multifactorial pathogenesis of autoimmune gastritis (AIG), organized into four interconnected modules. **(A)** Genetic Susceptibility: Specific HLA alleles (HLA-DRB1*03:01 and HLA-DRB1*04:05) and non-HLA genes (PTPN22, CTLA4) facilitate aberrant antigen presentation and immune dysregulation in genetically predisposed individuals. **(B)** Environmental Triggers: Helicobacter pylori infection can initiate autoimmunity through molecular mimicry, whereby bacterial antigens structurally resemble the gastric H+/K+-ATPase proton pump, leading to cross-reactive immune activation. **(C)** Autoimmune Response: Autoreactive CD4+ T helper cells (Th1 and Th17) are activated and drive both cellular and humoral immunity. Th1/Th17 cells produce pro-inflammatory cytokines (IFN-γ and IL-17), while providing help to autoreactive B cells, which differentiate into plasma cells producing anti-parietal cell antibodies (PCA) and anti-intrinsic factor antibodies (IFA). This culminates in the destruction of gastric parietal cells targeting the H+/K+-ATPase. **(D)** Clinical Consequences: Progressive parietal cell loss leads to achlorhydria, reduced intrinsic factor production, vitamin B12 malabsorption, and compensatory hypergastrinemia, ultimately increasing the risk of type 1 gastric neuroendocrine tumors (gNETs) and gastric adenocarcinoma. HLA, human leukocyte antigen; PTPN22, protein tyrosine phosphatase non-receptor type 22; CTLA4, cytotoxic T-lymphocyte-associated protein 4; IFN-γ, interferon-gamma; IL-17, interleukin-17; PCA, anti-parietal cell antibodies; IFA, anti-intrinsic factor antibodies; gNETs, gastric neuroendocrine tumors.

The pathogenesis of AIG is a complex, multifactorial process driven by a loss of immune tolerance to gastric parietal cell antigens in genetically susceptible individuals. This process involves a sophisticated interplay between genetic factors, environmental triggers, and a cascade of cellular and humoral immune responses that ultimately culminates in the destruction of the gastric oxyntic mucosa (**Figure 2**).

### The central role of autoreactive T cells

The primary effectors of parietal cell destruction in AIG are autoreactive CD4+ T helper (Th) cells, particularly Th1 and Th17 cells, which recognize the alpha and beta subunits of the gastric H+/K+-ATPase (the proton pump) as their target autoantigen (18). The autoimmune cascade is initiated when professional antigen-presenting cells, such as dendritic cells (DCs), capture H+/K+-ATPase released from damaged parietal cells and migrate to the gastric draining lymph nodes to prime naive CD4+ T cells via MHC class II presentation (3, 19).

Upon activation, these naive T cells differentiate into pathogenic Th1 and Th17 effector cells. Th1 cells produce pro-inflammatory cytokines, most notably interferon-gamma (IFN-γ), which activates macrophages and promotes a cell-mediated cytotoxic response (20).Th1-derived IFN-γ is thought to induce the aberrant expression of MHC class II molecules on gastric parietal cells, thereby facilitating antigen presentation and perpetuating the autoimmune response against the gastric mucosa (3). Simultaneously, Th17 cells, characterized by the production of interleukin-17 (IL-17), recruit neutrophils and other inflammatory cells to the gastric mucosa, further amplifying the inflammatory infiltrate and tissue damage (20). The balance between these effector T cells and regulatory T cells (Tregs), which normally function to suppress autoimmunity, is critically disrupted in AIG, favoring a pro-inflammatory state (21).

### Humoral immunity: the role of autoantibodies

While T cells are the primary drivers of tissue damage, the humoral immune system also plays a significant role, primarily through the production of characteristic autoantibodies. Activated Th cells provide help to autoreactive B cells, which then differentiate into plasma cells and produce anti-parietal cell antibodies (PCA) and anti-intrinsic factor antibodies (IFA) (11).

PCA, which target the H+/K+-ATPase, are found in 85-90% of AIG patients and serve as a key serological marker. Although their direct pathogenic role in parietal cell destruction is debated, they are believed to contribute to the inflammatory process through complement activation and antibody-dependent cell-mediated cytotoxicity (ADCC) (12, 22). IFA, on the other hand, are directed against intrinsic factor, a glycoprotein secreted by parietal cells that is essential for the absorption of vitamin B12 in the terminal ileum. IFA are highly specific for AIG and pernicious anemia. They are classified into two types: Type I (blocking antibodies) prevent the binding of vitamin B12 to intrinsic factor, while Type II (binding antibodies) bind to the intrinsic factor-B12 complex, preventing its absorption (23). IFA are highly specific diagnostic markers for pernicious anemia, the primary autoimmune cause of vitamin B12 deficiency (24).

### Genetic susceptibility: HLA and non-HLA genes

The familial clustering of AIG, as exemplified by the cases presented, strongly suggests a significant genetic component. The strongest genetic association is found within the human leukocyte antigen (HLA) complex on chromosome 6 (25). Specifically, the HLA-DRB1*03 and HLA-DRB1*04 alleles have been consistently linked to an increased risk of AIG in various populations (19, 26). These HLA-DR molecules are critical for presenting the H+/K+-ATPase peptides to CD4+ T cells, and specific alleles may have a higher binding affinity for these self-peptides, thus facilitating the activation of autoreactive T cells.

In addition to HLA genes, several non-HLA genes involved in immune regulation have been associated with AIG, contributing to a general predisposition to autoimmunity. Polymorphisms in the PTPN22 gene, which encodes a lymphoid tyrosine phosphatase that negatively regulates T-cell activation, are associated with AIG and a host of other autoimmune diseases (27). Similarly, variants in CTLA4, a key negative regulator of T-cell co-stimulation, and IL10, an anti-inflammatory cytokine, have also been implicated (26). Mouse models have been instrumental in identifying other potential susceptibility loci, such as Gasa1 and Gasa2 on chromosome 4, and Gasa3 on chromosome 6, although their direct correlates in human AIG are still under investigation (25).

Recent advances in immunological research have further elucidated the role of innate immunity in AIG pathogenesis. Beyond adaptive immune mechanisms, the innate immune system, including pattern recognition receptors (PRRs) and complement pathways, plays a crucial role in initiating and perpetuating the autoimmune response against gastric parietal cells (28). Toll-like receptors (TLRs), particularly TLR2 and TLR4, have been shown to be upregulated in the gastric mucosa of AIG patients and may contribute to the activation of dendritic cells and the subsequent priming of autoreactive T cells (19). Additionally, the complement system, particularly the alternative pathway, has been implicated in the pathogenesis of AIG, with evidence suggesting that complement-mediated tissue damage contributes to parietal cell destruction (29).The role of the gut microbiota in AIG has emerged as an important area of investigation. Dysbiosis, characterized by alterations in the composition and diversity of the intestinal bacterial community, has been documented in AIG patients and may contribute to disease pathogenesis through multiple mechanisms. These include increased intestinal permeability (“leaky gut”), translocation of bacterial lipopolysaccharides (LPS), and alterations in the production of short-chain fatty acids (SCFAs), which are important for maintaining immune homeostasis (7). The dysbiosis-associated increase in LPS translocation can activate TLRs and promote a pro-inflammatory state, potentially exacerbating the autoimmune response. Conversely, reduced production of butyrate-producing bacteria may impair the differentiation and function of regulatory T cells (Tregs), which are critical for maintaining immune tolerance (30). These findings suggest that microbiome-targeted interventions, such as fecal microbiota transplantation (FMT) or targeted probiotic therapy, may represent novel therapeutic approaches for AIG.

### The thyroid-gastric syndrome and polyglandular autoimmunity

The co-occurrence of AIG and AITD, known as the thyroid-gastric syndrome, is a well-established clinical entity (31), first described in the 1960s (32). As demonstrated in the illustrative cases, patients with AIG have a high prevalence of anti-thyroid antibodies (anti-TPO and anti-thyroglobulin), and conversely, patients with AITD have an increased risk of developing AIG (33). This association is attributed to shared genetic susceptibility factors (e.g.,HLA-DRB1*03:01) and common immunological pathways (34).

The thyroid-gastric syndrome is often considered a manifestation of autoimmune polyglandular syndrome (APS) type 3, which is characterized by the presence of AITD in combination with other organ-specific autoimmune diseases, such as AIG, type 1 diabetes, or vitiligo (33). The recognition of this syndrome has important clinical implications, as it underscores the need for systematic screening for associated autoimmune conditions in patients diagnosed with either AIG or AITD (35).

### Environmental triggers and the role of *H. pylori*

The relationship between AIG and Helicobacter pylori infection is complex. H. pylori infection can trigger an autoimmune response through molecular mimicry, where bacterial antigens share structural similarities with host proteins, such as the H+/K+-ATPase proton pump (19). This can lead to the development of AIG in a subset of genetically susceptible individuals.

Conversely, the chronic atrophic gastritis and achlorhydria characteristic of AIG create an unfavorable environment for H. pylori colonization, which may explain the low prevalence of active H. pylori infection in patients with established AIG (36). However, a history of past H. pylori infection may still be relevant to the pathogenesis of AIG and the risk of gastric cancer (37).

## Unresolved questions and controversies

Despite significant advances in understanding the immunopathogenesis of AIG, several frontier controversies remain unresolved. A major area of debate centers on the specific intervention strategies for regulating the Th17/Treg balance. While preclinical models suggest that restoring this balance—such as through STAT3 inhibition (21)—can mitigate experimental autoimmune gastritis by reducing inflammation and limiting early metaplastic changes, translating these findings into clinical practice remains challenging. Currently, there are no specific, targeted immunomodulatory therapies approved for restoring Treg function in AIG patients. Furthermore, the reversibility of AIG following *Helicobacter pylori* eradication remains a subject of intense controversy. While some studies suggest that early eradication may halt or even reverse the autoimmune cascade triggered by molecular mimicry, others argue that once the autoreactive T-cell response is fully established, the progression of oxyntic atrophy becomes independent of the bacterial infection. These unresolved issues highlight the critical need for longitudinal studies to determine the optimal therapeutic window for immunomodulation and to clarify the long-term impact of *H. pylori* eradication on the natural history of AIG.

## Diagnostic approaches and challenges

**Figure 3:**
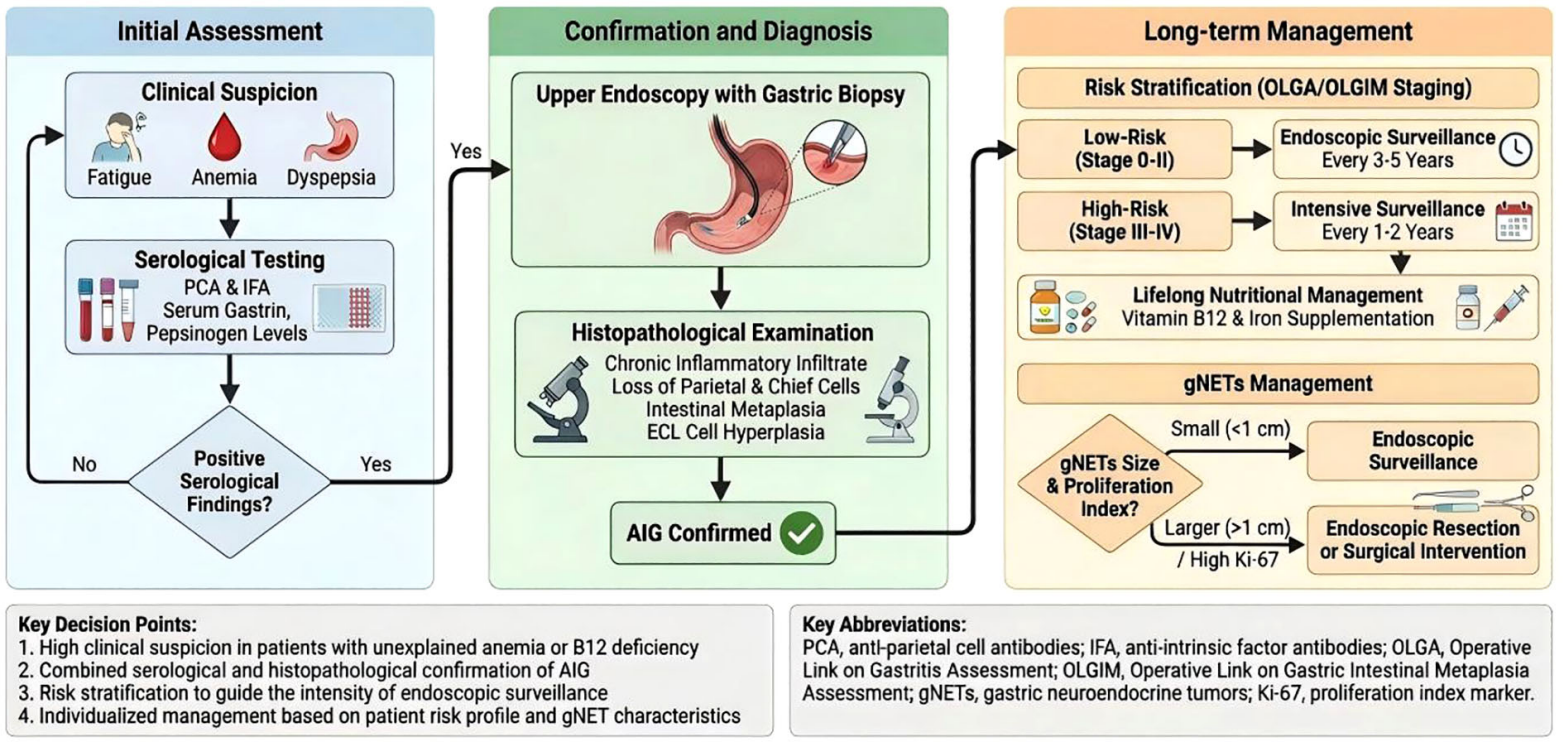
Diagnostic and management algorithm for autoimmune gastritis. This flowchart outlines the comprehensive clinical pathway for the diagnosis and management of AIG, organized into three sequential phases. Phase 1 — Initial Assessment: Clinical suspicion is raised in patients presenting with unexplained fatigue, anemia, or dyspepsia. Serological testing includes PCA, IFA, serum gastrin, and pepsinogen levels. Patients with positive serological findings proceed to endoscopic evaluation; those with negative results but high clinical suspicion should undergo repeat testing after 12–24 months. Phase 2 — Confirmation and Diagnosis: Upper endoscopy with gastric biopsy is performed following the Updated Sydney System protocol (minimum five biopsies: two antrum, one incisura, two corpus). Histopathological examination confirms AIG based on chronic inflammatory infiltrate, loss of parietal and chief cells, intestinal metaplasia, and ECL cell hyperplasia. Phase 3 — Long-term Management: Risk stratification is based on OLGA/OLGIM staging. Low-risk patients (Stage 0–II) undergo endoscopic surveillance every 3–5 years, while high-risk patients (Stage III–IV) require intensive surveillance every 1–2 years. Lifelong nutritional management (vitamin B12 and iron supplementation) is recommended for all patients. For type 1 gNETs, management is guided by tumor size and proliferation index (Ki-67): small tumors (<1 cm) are managed with endoscopic surveillance, while larger tumors (>1 cm) or those with high Ki-67 require endoscopic resection or surgical intervention. Abbreviations: PCA, anti-parietal cell antibodies; IFA, anti-intrinsic factor antibodies; OLGA, Operative Link on Gastritis Assessment; OLGIM, Operative Link on Gastric Intestinal Metaplasia Assessment; gNETs, gastric neuroendocrine tumors; Ki-67, proliferation index marker; ECL, enterochromaffin-like.

The diagnosis of AIG requires a high index of suspicion and a multi-pronged approach that integrates clinical presentation, serological markers, and endoscopic and histopathological findings. The often insidious onset and non-specific symptoms, such as dyspepsia, fatigue, and anemia, make the diagnosis challenging (**Figure 3**).

### Serological diagnosis: a non-invasive first step

Serological testing is the cornerstone of non-invasive screening and diagnosis of AIG. The primary markers are autoantibodies and biomarkers of gastric function.

### Autoantibody testing

#### Anti-parietal cell antibodies

Targeting the H+/K+-ATPase, PCA are detected in approximately 85-90% of AIG patients, making them a sensitive marker (38). However, their specificity is limited, as they can be found in other autoimmune diseases (e.g., type 1 diabetes, AITD), in H. pylori gastritis, and in 7.8-19.5% of healthy individuals (39). Therefore, a positive PCA test alone is not sufficient for diagnosis.

#### Anti-intrinsic factor antibodies

These antibodies are highly specific for AIG (95-100%) but have a lower sensitivity (50-70%), particularly in the early stages of the disease (15, 38). A positive IFA test is highly suggestive of AIG and supports the diagnosis when combined with clinical and endoscopic findings, but a negative result does not rule it out. The illustrative cases presented earlier demonstrate this variability, with one patient being IFA positive but PCA negative, highlighting the importance of testing for both antibodies to maximize diagnostic yield (15).

### Gastric function biomarkers

#### Pepsinogen I and II

Pepsinogen I (PGI) is secreted by chief cells in the gastric body and fundus, while pepsinogen II (PGII) is secreted throughout the stomach (40). In AIG, the autoimmune destruction of parietal cells in the oxyntic mucosa leads to hypochlorhydria and subsequent loss of chief cells, resulting in a marked decrease in PGI, while PGII levels (secreted throughout the stomach) are relatively preserved, leading to a characteristic low PGI/PGII ratio (41).

#### Gastrin-17

In response to achlorhydria, G-cells in the gastric antrum are stimulated to produce excess gastrin, leading to hypergastrinemia (7, 42). Marked hypergastrinemia (elevated fasting serum gastrin levels) is a hallmark of AIG (43).

### Endoscopic and histopathological diagnosis

Upper endoscopy with biopsy is the gold standard for confirming the diagnosis of AIG and assessing the extent of gastric atrophy and the presence of pre-neoplastic lesions (44).

### Endoscopic findings

Endoscopic features of AIG can be subtle in the early stages but become more apparent as the disease progresses. Classic findings, often referred to as the “reverse atrophy pattern,” include pallor and thinning of the gastric rugae in the corpus and fundus, contrasting with a relatively spared antrum (45, 46). Intestinal metaplasia may appear as whitish, slightly elevated plaques. In advanced stages, the gastric body may appear smooth and featureless, a finding known as “bald fundus” (11).

### Histopathological evaluation

Histopathological examination of biopsies from the gastric corpus and antrum is essential. To ensure accurate diagnosis and risk stratification, endoscopic surveillance must adhere to standardized biopsy protocols. According to the Updated Sydney System (47) and the MAPS II guidelines, a minimum of five biopsies should be obtained: two from the antrum (greater and lesser curvature), one from the incisura angularis, and two from the corpus (greater and lesser curvature). In the specific context of suspected AIG, particular attention must be paid to obtaining adequate samples from the oxyntic mucosa of the corpus and fundus, as well as targeting any visible focal lesions or areas of suspected metaplasia. The characteristic findings in the corpus include a diffuse, chronic inflammatory infiltrate composed of lymphocytes and plasma cells, a marked loss of parietal and chief cells, and their replacement by mucous-secreting cells (pseudopyloric metaplasia) or intestinal-type epithelium (intestinal metaplasia) (11). Enterochromaffin-like (ECL) cell hyperplasia, a precursor to gNETs, is also a common finding (48).

### Diagnostic challenges and pitfalls

#### Seronegative AIG

A subset of patients with histologically confirmed AIG may be negative for both PCA and IFA, posing a diagnostic challenge. In these cases, the diagnosis relies on a high index of clinical suspicion and typical endoscopic and histopathological findings (49).

#### “Macro B12” phenomenon

The presence of “macro B12,” a complex of vitamin B12 and an autoantibody (often an immunoglobulin), can lead to falsely normal or elevated serum B12 levels, masking an underlying deficiency. In patients with suspected AIG and discordant clinical and laboratory findings, measuring methylmalonic acid (MMA) and homocysteine levels can help unmask a functional B12 deficiency (50).

A systematic diagnostic algorithm, often based on international consensus guidelines (38), has been proposed to optimize the diagnosis of AIG in clinical practice. This algorithm integrates clinical suspicion (based on symptoms and risk factors), serological screening (PCA and IFA), gastric function biomarkers (pepsinogen I/II ratio and gastrin-17), and confirmatory endoscopic and histopathological findings. Initial screening should include both PCA and IFA testing, as their combined use maximizes diagnostic sensitivity and specificity (7). Patients with positive serology should proceed to upper endoscopy with targeted biopsies from the corpus and antrum to assess the extent of atrophy and the presence of pre-neoplastic lesions. In cases of serological discordance (e.g., positive serology but normal endoscopy, or vice versa), repeat testing and follow-up endoscopy after 12–24 months are recommended to capture the progressive nature of the disease (38, 51). This structured approach not only improves diagnostic accuracy but also facilitates early identification of high-risk patients who require more intensive surveillance and potential immunomodulatory interventions.

## Limitations of current diagnostic approaches and emerging diagnostic technologies

While serological testing and endoscopic-histopathological examination remain the gold standard for AIG diagnosis, several limitations warrant discussion. First, approximately 5-10% of patients with histologically confirmed AIG are seronegative for both PCA and IFA, making diagnosis challenging and relying heavily on clinical suspicion and endoscopic findings (49). Second, the dynamic nature of autoantibodies, including potential seroconversion events, may necessitate serial testing in suspected cases. Third, the non-specific nature of pepsinogen I/II ratios and gastrin-17 levels limits their utility as standalone diagnostic markers, as these biomarkers can be abnormal in other conditions affecting gastric function (52).

To address these limitations, several emerging diagnostic technologies are being investigated. Artificial intelligence (AI)-assisted endoscopy, utilizing deep learning algorithms trained on large endoscopic image databases, has shown promising results in improving the detection of atrophic changes and dysplasia, with some studies reporting diagnostic accuracy comparable to or exceeding that of experienced endoscopists (53). Additionally, multi-omics approaches, including transcriptomics, proteomics, and metabolomics, are being explored to identify novel biomarkers for AIG diagnosis and risk stratification. For instance, serum proteomic signatures and specific microRNA profiles have been associated with disease activity and progression, and may eventually serve as non-invasive diagnostic and prognostic tools (54). While these emerging technologies are not yet ready for routine clinical practice, they represent promising avenues for improving the accuracy and efficiency of AIG diagnosis in the future.

## Risk stratification for gastric malignancies

AIG is a well-established pre-neoplastic condition, conferring an increased risk for both gastric adenocarcinoma and type 1 gNETs. Therefore, accurate risk stratification and long-term surveillance are critical components of management (37).

### Gastric adenocarcinoma

The risk of gastric adenocarcinoma in AIG patients is estimated to be 3–5 times higher than in the general population, although recent meta-analyses suggest this risk may be substantially greater, particularly in patients with pernicious anemia (37, 55). The development of cancer follows the Correa cascade, a multi-step process that progresses from chronic inflammation to atrophic gastritis, intestinal metaplasia, dysplasia, and finally, carcinoma. The extent and severity of atrophic gastritis and intestinal metaplasia are key determinants of cancer risk (56).

### OLGA/OLGIM staging systems

The Operative Link on Gastritis Assessment (OLGA) and Operative Link on Gastric Intestinal Metaplasia Assessment (OLGIM) staging systems are histopathological tools used to stratify the risk of gastric cancer (57). These systems combine the topographical location (antrum vs. corpus) and severity of atrophy (OLGA) or intestinal metaplasia (OLGIM) to generate a score from 0 to IV. Patients with high-risk stages (OLGA/OLGIM III-IV) have a significantly increased risk of developing gastric cancer and require more intensive endoscopic surveillance (58, 59).

### Gastric neuroendocrine tumors

Type 1 gNETs are the most common neoplastic complication of AIG, occurring in up to 10% of patients. They arise from the hyperplasia of ECL cells, which is driven by the trophic effect of chronic hypergastrinemia. Most type 1 gNETs are small, multiple, and have a low malignant potential (1). However, larger tumors (>1 cm) or those with a high proliferation index (Ki-67 >2%) have a higher risk of metastasis and require more aggressive management (60).

### Molecular biomarkers for risk stratification

Recent advances in molecular pathology have identified several potential biomarkers for risk stratification in AIG. For example, aberrant DNA methylation patterns and specific microRNA profiles have been associated with an increased risk of progression to dysplasia and carcinoma (61). Machine learning models integrating clinical, serological, and molecular data are also being developed to create more precise, individualized risk prediction tools (62).

### Prognostic prediction models and individualized management strategies

Recent advances in risk stratification have led to the development of multi-parameter prognostic models that integrate clinical, serological, endoscopic, and molecular data to predict disease progression and malignancy risk. Risk stratification models for AIG increasingly integrate multi-parameter data, combining histological staging (OLGA/OLGIM) with serological markers (pepsinogen I and gastrin-17) to enhance predictive accuracy for gastric neoplasia (58).

Based on prognostic risk stratification, individualized management strategies can be tailored to patient risk profiles. Low-risk patients (OLGA/OLGIM stage 0-II, no dysplasia, normal pepsinogen I levels) may be managed with standard nutritional supplementation and endoscopic surveillance every 5 years, whereas high-risk patients (OLGA/OLGIM stage III-IV, presence of dysplasia, low pepsinogen I levels) warrant more intensive surveillance every 1–2 years and may be candidates for early immunomodulatory interventions (8, 58). Emerging evidence suggests that early immunomodulation with corticosteroids or other immunosuppressants in patients with highly inflammatory early-stage disease may slow disease progression and reduce the incidence of gastric cancer, although large-scale randomized controlled trials are needed to confirm this hypothesis (63). Additionally, the identification of specific molecular subtypes of AIG based on gene expression or epigenetic profiles may enable truly personalized medicine approaches, allowing clinicians to predict treatment response and select optimal therapeutic strategies for individual patients.

## Management and emerging therapeutic strategies

The management of AIG is multifaceted, focusing on correcting nutritional deficiencies, endoscopic surveillance for malignancies, and, in select cases, addressing the underlying autoimmune process.

### Correction of nutritional deficiencies

#### Vitamin B12

Lifelong parenteral (intramuscular) or high-dose oral vitamin B12 supplementation is essential for all patients with pernicious anemia and for those with evidence of B12 deficiency without anemia (38).

#### Iron

Iron deficiency is also common in AIG, resulting from achlorhydria which impairs the conversion of dietary ferric iron (Fe3+) to the more readily absorbed ferrous form (Fe2+). Oral iron supplementation, often in combination with vitamin C to enhance absorption, is the first-line treatment (7, 64).

### Endoscopic surveillance

Regular endoscopic surveillance is recommended for all AIG patients to detect dysplasia and early-stage gastric cancer. The frequency of surveillance is tailored to the individual patient’s risk profile, as determined by the OLGA/OLGIM stage (9). Patients with advanced stages (III/IV) are generally recommended for endoscopic surveillance every 3 years, while those with lower risk may be monitored less frequently (9, 44).

### Management of treatment failures and complications

Iron deficiency anemia, another common complication, occurs in 20-30% of AIG patients due to reduced gastric acid and impaired iron absorption. Iron supplementation should be optimized, and in refractory cases, intravenous iron therapy may be considered (1, 64). Additionally, patients may develop neurological complications from prolonged B12 deficiency, including subacute combined degeneration (SCD) of the spinal cord, which can be irreversible if not treated promptly (65). Early recognition and aggressive management of B12 deficiency are therefore critical to prevent these serious neurological sequelae.

Despite optimal management, some AIG patients experience treatment failures or develop serious complications that require specialized interventions. B12 supplementation failure, defined as persistent B12 deficiency despite adequate supplementation, occurs in 10-15% of patients and may be due to concurrent gastrointestinal disorders such as celiac disease, small intestinal bacterial overgrowth (SIBO), or pancreatic insufficiency (66). In such cases, investigation for these comorbidities is essential, and higher doses or more frequent B12 supplementation (e.g., weekly or bi-weekly intramuscular injections) may be necessary (67).

Long-term follow-up studies confirm that AIG is associated with an increased risk of mortality, primarily driven by neoplastic complications such as gastric cancer and gastric neuroendocrine tumors, as well as severe B12 deficiency-related neurological sequelae (7). These findings underscore the importance of comprehensive, multidisciplinary management and close long-term follow-up to optimize patient outcomes and quality of life.

### Management of gNETs

The management of type 1 gNETs depends on their size, number, and proliferation index. Small, asymptomatic tumors (<1 cm) can often be managed with endoscopic surveillance (68). Larger or symptomatic tumors often necessitate endoscopic resection. For patients with recurrent or numerous Type 1 gNETs refractory to other treatments, surgical intervention, such as antrectomy, may be considered to normalize gastrin levels and prevent further tumor development (69).

### Emerging therapeutic strategies

While current management focuses on the consequences of AIG, several emerging therapies aim to target the underlying autoimmune process.

#### Immunomodulatory therapies

While standard management for autoimmune atrophic gastritis focuses on vitamin B12 supplementation, limited case reports and mechanistic data suggest that corticosteroids or other immunosuppressants might offer benefits, particularly in early, highly inflammatory stages of the disease(level of evidence: preclinical and case reports) (1, 70). However, therapeutic options remain limited, with only the gastrin receptor antagonist Netazepide having reached clinical trials, while broader immunomodulatory strategies are still in the preclinical stage(level of evidence: phase II RCT) (3).

#### Microbiota dysbiosis and immune tolerance in AIG

The role of the gut microbiota in AIG pathogenesis has emerged as a critical area of investigation, with dysbiosis being increasingly recognized as both a consequence and a potential driver of disease progression. Metagenomic and metabolomic analyses of AIG patients have revealed significant alterations in microbial composition compared to healthy controls, characterized by reduced microbial diversity, decreased abundance of beneficial commensals (particularly butyrate-producing bacteria such as Faecalibacterium prausnitzii and Roseburia spp.), and increased abundance of potentially pathogenic species (71). These dysbiotic changes are associated with increased intestinal permeability, enhanced bacterial lipopolysaccharide (LPS) translocation, and elevated circulating LPS levels, which can activate pattern recognition receptors on innate immune cells and perpetuate a pro-inflammatory state (72).Mechanistically, dysbiosis impairs the production of short-chain fatty acids (SCFAs), particularly butyrate, which are crucial metabolites for maintaining immune homeostasis. Butyrate serves as a histone deacetylase inhibitor and promotes the differentiation and expansion of regulatory T cells (Tregs) through GPR43 and GPR109a signaling, thereby enhancing immune tolerance and suppressing pro-inflammatory responses (73). In AIG patients, the reduced abundance of butyrate-producing bacteria correlates with decreased fecal butyrate levels and impaired Treg function, suggesting a mechanistic link between dysbiosis and autoimmune activation. Conversely, interventions aimed at restoring beneficial microbiota, such as targeted probiotic therapy with specific strains (e.g., Faecalibacterium prausnitzii) or fecal microbiota transplantation (FMT), have shown promise in preclinical and early clinical studies in restoring immune tolerance and reducing disease activity (74). These findings suggest that microbiota-directed therapies may represent a novel and potentially disease-modifying approach for AIG, particularly when combined with conventional management strategies.

#### Proposed research framework for microbiome-targeted therapy

Given the limited clinical translational data currently available for microbiome interventions in AIG, future research must transition from observational studies to rigorous, hypothesis-driven clinical trials. We propose a structured research framework to evaluate the efficacy of microbiome-targeted therapies. First, longitudinal cohort studies are needed to dynamically monitor the gastric and gut microbiome across different OLGA/OLGIM stages, identifying specific microbial signatures associated with disease progression (71). Second, well-designed randomized controlled trials (RCTs) evaluating targeted probiotics (e.g., Faecalibacterium prausnitzii) or fecal microbiota transplantation (FMT) should be initiated (74). These trials must establish strict inclusion criteria, focusing on early-stage AIG patients with active inflammation rather than end-stage atrophy, and utilize robust primary endpoints, such as histological improvement (reduction in inflammatory infiltrate) and restoration of the Th17/Treg balance in the gastric mucosa (73). Finally, integrating multi-omics approaches—combining metagenomics with host transcriptomics and metabolomics—will be essential to elucidate the precise molecular mechanisms by which microbial metabolites, such as short-chain fatty acids, modulate immune tolerance in the gastric microenvironment (73). In summary, microbiome-targeted interventions—including targeted probiotics and FMT—represent promising but still investigational therapeutic approaches for AIG, and their clinical efficacy awaits validation through the rigorous trial designs proposed above (level of evidence: preclinical and early clinical studies) (74).

#### Acid replacement therapy

The use of betaine hydrochloride to restore gastric acidity is a controversial but intriguing concept. By normalizing the gastric pH, this approach may help improve nutrient absorption and potentially modulate the gut microbiome, although robust clinical evidence is still lacking(level of evidence: case series and expert opinion) (75).

#### The role of proton pump inhibitors

The use of proton pump inhibitors (PPIs) in patients with AIG presents a unique clinical paradox. AIG is inherently characterized by hypochlorhydria or achlorhydria due to parietal cell destruction; therefore, the routine use of acid-suppressive therapy is generally not recommended and may exacerbate the existing physiological deficit (76). Furthermore, long-term PPI use independently induces hypergastrinemia and can promote enterochromaffin-like (ECL) cell hyperplasia, potentially compounding the risk of type 1 gastric neuroendocrine tumors already present in AIG patients (1). However, PPIs may still be cautiously considered in specific, limited clinical scenarios, such as the short-term management of concurrent severe reflux esophagitis or the healing of peptic ulcers, provided that the benefits outweigh the risks of exacerbating hypergastrinemia. In such cases, the lowest effective dose should be used for the shortest possible duration, accompanied by close endoscopic surveillance (76).

#### Stem cell therapy

Preclinical research, particularly utilizing Mesenchymal Stem Cells (MSCs) and gastric organoids, suggests potential for regenerating damaged gastric mucosa and restoring function(level of evidence: preclinical animal models) (77). It must be emphasized that these approaches remain investigational. While still in its infancy, this represents a promising avenue for future therapeutic development (78).

### AIG in pediatric populations

Although AIG is predominantly diagnosed in older adults, its occurrence in pediatric populations is increasingly recognized, albeit rare. Pediatric AIG often presents with significant clinical heterogeneity, frequently manifesting as refractory iron deficiency anemia rather than the classic pernicious anemia seen in adults (79). Furthermore, early-onset AIG is strongly associated with other autoimmune conditions, particularly type 1 diabetes mellitus and autoimmune thyroid disease, underscoring the importance of screening for gastric autoimmunity in children presenting with these endocrinopathies (80). The diagnosis in children is challenging due to the scarcity of specific guidelines and the reluctance to perform invasive endoscopic evaluations (81). However, early identification is crucial, as the prolonged disease duration in pediatric-onset AIG may theoretically increase the lifetime risk of neoplastic complications. Future international, multicenter registries are urgently needed to better characterize the natural history, optimize diagnostic algorithms, and establish evidence-based surveillance protocols specifically tailored for pediatric AIG patients (80).

## Conclusion and future perspectives

Autoimmune gastritis is a complex and often underdiagnosed condition with significant long-term clinical implications. A deeper understanding of its immunopathogenesis has led to improved diagnostic strategies and the development of more precise risk stratification models. The recognition of AIG as a systemic disease, often co-occurring with other autoimmune conditions, underscores the importance of a holistic and multidisciplinary approach to patient care.

The evolving understanding of AIG as a systemic autoimmune disease with significant extraintestinal manifestations has important implications for clinical practice. Future research should prioritize (1): the development of non-invasive biomarkers for early diagnosis and disease monitoring (2); the validation of multi-parameter prognostic models in diverse populations (3); large-scale clinical trials of emerging immunomodulatory and microbiome-targeted therapies,with specific attention to the structured research framework proposed herein for microbiome-targeted interventions (4); the integration of patient-reported outcomes and quality of life measures into clinical trials and routine practice. By advancing our understanding of AIG and implementing precision medicine approaches, we can improve early diagnosis, optimize individualized management, and ultimately reduce the burden of this complex autoimmune disease.
